# In sickness and health: A reflexive thematic analysis of ethical considerations and experiences of owners of cats treated for diabetes mellitus

**DOI:** 10.1371/journal.pone.0341759

**Published:** 2026-02-02

**Authors:** Ninni Rothlin-Zachrisson, Bodil Ström Holst, Malin Öhlund, Janeth Leksell, Helena Röcklinsberg

**Affiliations:** 1 Department of Clinical Sciences, Swedish University of Agricultural Sciences, Uppsala, Sweden; 2 Swedish Medical Products Agency, Uppsala, Sweden; 3 Department of Medical Sciences, Uppsala University, Uppsala, Sweden; 4 Department of Animal Science and Welfare, Swedish University of Agricultural Sciences, Uppsala, Sweden; Universidade de Trás-os-Montes e Alto Douro: Universidade de Tras-os-Montes e Alto Douro, PORTUGAL

## Abstract

Cats are widely regarded as beloved family members, with whom owners develop strong emotional bonds. When a cat is diagnosed with diabetes mellitus, many owners choose to pursue medical treatment despite the significant commitment and resources required for disease management. This study qualitatively explored the factors influencing treatment decisions for cats diagnosed with diabetes mellitus, focusing on the emotional, practical, and ethical challenges that shape owners’ choices. In-depth interviews were conducted with 12 cat owners in Sweden, all of whom had opted to initiate treatment for their cats diagnosed with diabetes mellitus. The data were systematically analysed using reflexive thematic analysis, generating three themes within an overarching theme encapsulating key aspects of cat ownership. These themes centered on owners’ perceived responsibility of care, maintaining the cats’ quality of life, and the burden of end-of-life decisions. Findings highlight the interplay of emotional attachment, caregiving responsibilities, and ethical considerations in decision-making. We argue that recognising the emotional bonds between owners and their cats and providing personalised care plans that balance medical needs with owners’ life situations can improve communication, veterinary support and cat welfare. Addressing both the medical and emotional aspects of caregiving is crucial for fostering a sustainable and positive caregiving experience.

## Introduction

Cats hold a unique place in human households, often regarded as beloved family members [1–6] with whom owners develop strong emotional bonds [7]. This can result in a significant emotional investment, especially if the cat becomes ill. Advanced treatment options exist and are constantly developing, allowing for the management of many acute and chronic diseases. Diabetes mellitus (DM) presents a particularly relevant case in this context. Unlike some other chronic conditions, management of DM in cats typically involves daily hands-on care, including insulin injections and regular blood glucose monitoring [8,9]. The commitment needed for treatment can transform owners’ everyday lives, reshaping routines and creating new caregiving responsibilities [10]. Applying alternative, less labour-intensive treatment strategies (e.g., refraining from home blood glucose monitoring) can provide greater flexibility for owners who may find a more intense regimen difficult to maintain, although these modified protocols may decrease the likelihood of achieving successful treatment outcomes [11–13]. A proportion of cats with DM may achieve diabetic remission, defined as the cat maintaining euglycaemia after discontinuation of exogenous hypoglycaemic treatment [14], but many cats require lifelong insulin treatment to maintain a stable condition.

DM is a chronic, but often manageable disease. The success of treatment relies heavily on the owners commitment and resources. The decision to medically treat the cat diagnosed with DM is not always straightforward, and one of ten cats is euthanised upon diagnosis [15]. Some aspects as to why owners may choose not to treat their cats have been explored, highlighting factors such as financial constraints, perceived decreased quality of life for the cat, and the time demands associated with an intensive treatment regime and care for the cat [15–17]. Less research has been conducted on the reasons behind an owner’s choice to initiate and maintain treatment for their cats, rather than deciding upon euthanasia. Such research is important, because understanding the motivations behind these decisions can offer deeper insights into how owners perceive their responsibilities toward their cats in the context of chronic illnesses, thereby facilitating better communication between veterinarians and owners [18,19].

This study aims to deepen the understanding of how owners manage the emotional, practical, and ethical challenges of treating DM in their cats. It examines the motivations behind treatment decisions, the impact of the cat’s dependency, and how owners approach end-of-life decisions. By exploring these factors, the study addresses the relationship between emotional bonds, caregiving responsibilities, and ethical considerations, offering insights to improve veterinary support and owner guidance.

## Methodology

Data were generated through individual in-depth interviews with 12 owners of cats diagnosed with DM, all of whom had chosen to initiate medical treatment following their cat’s DM diagnosis independent of this study. Prior to the start of the study, an ethical review was conducted by the Swedish Ethical Review Authority (Dnr 2021−00939), including approval of video and audio recordings. See S1 Appendix for a detailed description of methodological considerations and reflexivity.

### Recruitment process and participants

Recruitment for the study involved a combination of purposive sampling and convenience sampling [20]. Nine of the cat owners were recruited by posting a call for participants in a Facebook group with over 3900 members, previously created by the research team for individuals (both cat owners and veterinary professionals) interested in DM in cats. Although owners may be referred to the group regardless of their level of commitment, online communities may attract more highly motivated owners. In an effort to diversify perspectives and values, two additional cat owners were directly approached by the primary author in the Facebook group. One owner was recruited by personal contacts. The research group also attempted to include owners encountered during visits to veterinary clinics; however, no owners were recruited in this way, possibly due to time constraints in the clinical setting or to avoid placing an additional informational burden on owners who were already receiving extensive input during the consultation. During the recruitment process, which began on 13 November 2023 and concluded on 22 February 2024, information about the research project, including the primary author, was provided.

Owners expressing interest in participation were asked to provide information regarding the status of their cat (alive or deceased), the type of treatment (no treatment initiated, insulin injections, dietary changes, or other), and whether the cat had achieved remission. This information served as a screening mechanism to ensure diversity among owner experiences. The inclusion criteria further included residency in Sweden, and current or past ownership of one or more cats diagnosed with DM. Participation was voluntary, no compensation was offered, and each informant received written information about the study and data management, as well as an informed consent form including information about the possibility to withdraw from the study without explanation at any time.

A total of 12 owners was interviewed, the majority of whom (11/12) were female. Three owners lived in the countryside and nine lived in towns. Participants came from different geographic regions of Sweden. All 12 cats had been treated with insulin, and three had experienced remission on at least one occasion. At the time of the interviews, three cats were no longer alive: two had been euthanised due to DM, and one due to non–diabetes-related issues. Four owners had prior experience with DM in humans (n = 3) and in cats (n = 1) before their cats were diagnosed. For an overview of owners’ and cats’ characteristics, see Table 1.

**Table 1:** An overview of the characteristics of the participants and their cats.

Participant*	Age** (years)	Gender	Previous experience of diabetes mellitus	Cat age at diagnosis (years)	Status of the cat at time of the interview
Marie	50	Female	No	4	Euthanised, previously treated with insulin
Amelia	40	Female	No	5	Euthanised, previously treated with insulin
Karin	50	Female	No	8	Ongoing insulin treatment
Fredrik	40	Male	Yes	9	Ongoing insulin treatment, relapsed from remission
Sofia	60	Female	Yes	7	Ongoing insulin treatment, relapsed from remission
Charlotta	60	Female	Yes	12	Ongoing insulin treatment
Mia	30	Female	No	5	Ongoing insulin treatment
Anna	50	Female	No	10	In remission, previously treated with insulin
Eva	40	Female	Yes	14	Ongoing insulin treatment
Ingrid	20	Female	No	7	Ongoing insulin treatment
Hanna	30	Female	No	5	Euthanised, previously treated with insulin
Julia	40	Female	No	12	Ongoing insulin treatment

*Pseudonym

**Approximated to nearest ten.

### Data collection

To obtain detailed descriptions and complexity of lived experiences, individual in-depth interviews were chosen. The interviews were conducted in Swedish, the majority through a digital video conference program (Zoom). After verbal consent was obtained from the interviewee and documented in writing by the interviewer, interviews were recorded for later transcription. One interview was conducted without video recording as desired by the participant. All interviews were performed by the primary author, with only the interviewee and the interviewer present. The interviews were semi-structured, originating from a predetermined guide (see S2 Appendix), and lasted approximately 50–60 minutes each. Most questions were open-ended, to minimise impact and so as not to interfere with interviewee opinions and experiences and were piloted on two other cat owners to ensure effectiveness. The transcripts were neither proofread nor corrected by the interviewees. However, interviewees were encouraged to reach out after the interviews if they had any questions, wished to clarify anything they had said, or wanted to withdraw their participation at any time. After each interview, initial thoughts and impressions were noted (field notes).

The number of participants needed was estimated stepwise and continuously and was adjusted during the course of data collection and initial data analysis, guided by the concept of information power [21] and Braun and Clarke’s reflections on data collection within RTA [22]. The dataset was reviewed throughout data generation by using field notes, reflecting on its diversity, on data quality and relevance to the research questions. After ten interviews, the dataset was judged to be sufficiently rich. However, given the explorative nature of the study, two additional interviews were conducted to avoid prematurely closing data collection and reduce the risk of limiting sample specificity regarding participants’ experiences. The recorded video interviews were transcribed manually and verbatim by the first author using the software oTranscribe. The average word length for the transcript data items was 6541 words (range 4740–8127). Pseudonyms were used for all interview participants during the analytical process and writing of the report.

### Analytical process and reflexivity

A six-phase process of inductive thematic analysis was undertaken with a focus on broad thematic patterning across the dataset, while acknowledging the impact of the researcher on both data collection and analysis and embracing researcher subjectivity as an asset [23,24]. Analysis was grounded in a constructivist paradigm and conducted applying an experiential orientation towards the data, acknowledging individual and diverse experiences and granting participants a central role in shaping the narrative. Different phases of analysis called for different data approaches, and the software Delve was used alongside hard copies of data extracts, with each step of the analysis clearly documented. The transcripts were iteratively and systematically worked through, first without particular focus, although preliminary observations were annotated. The generation of codes included both semantic and latent elements of the data reading. Interesting data excerpts relevant to the research questions were then coded, and after refinement, codes were sorted after patterns of shared meaning around owners’ motivations to treat, on ownership and how owners related to the cat as a receiver of care, and end-of-life decision-making. As analysis continued, it became apparent that ownership itself served as a broader conceptual framework encompassing reasoning across these areas. All data and codes related to these patterns were collected and sorted into candidate themes. Throughout the analytic process, themes were continually refined and reviewed against their underlying codes and supporting data extracts. During data collection and analysis, regular discussions within the research team served to draw upon the diverse perspectives and experiences, to enhance and broaden our analytical insights. To foster reflexivity and facilitate the discernment and contemplation of divergent viewpoints, two transcripts were independently coded by both the first author and a co-author. The reflexive process, which includes reflections on researcher positionality and how it may influence data engagement [24], was encouraged by ongoing reflection and discussions between co-authors, helping to reduce researcher preconceptions and to foster inclusivity in perspectives.

## Analysis and discussion

### Thematic structure

One overarching theme, *It’s for life: defining cat ownership*, was generated as the conceptual anchor for three themes. *In sickness and health* reflected owners’ considerations when initiating treatment for cats with DM, emphasizing the cat as a family member and the evolving human–animal relationship. *The cat: opportunities and responsibilities* was created from owners’ rationalizations regarding treatment, focusing on quality of life and compromises in medical care. *Who decides about life?* addressed euthanasia decisions, highlighting responsibility, inner conflict, and attempts to simplify the process. An overview of the analytical structure is provided in Table 2.

**Table 2:** Thematic structure, summaries of themes, and thematic connections in owner´s experiences of treating DM in cats.

Analytic structure:	Summary:	Examples of key data extracts:
**Overarching theme**		
It’s for life—defining cat ownership	Aspects that create and shape cat ownership and its implications.	Far-reaching responsibility: *“When you choose to become a cat owner, it is for life.”*
**Themes**		
In sickness and health	Owners’ sense of duty and motivation to treat their cats with diabetes mellitus.	The cat as a family member: *“She is my responsibility, like a child.”* Relationships matter: *“If I’d had her since she was a kitten, I would have felt differently.”*
The cat: opportunities and responsibilities	Adaptation of treatment routines to balance the cat’s quality of life and the owner’s daily life.	Pragmatic care: *“If it had been exactly 6 a.m. and 6 p.m., it wouldn´t have worked.”* Responsibilities and guilt: *“If I stay longer at a friend’s house, I feel guilty.”*
Who decides about life?	Ethical and emotional challenges in end-of-life decision-making.	A principled choice: *“I will never euthanise because of my energy or finances.”* Weight of the decision: *“I wish she would just fall asleep peacefully without [me] having to decide.*

### Overarching theme: It´s for life—defining cat ownership

The literature shows that a strong sense of ownership can lead to more responsible caretaking practices [25], although definitions of ownership may be interpreted differently. Also in this study, most interviewees placed an extensive emphasis on ownership, constituting a foundation for further discussions. Although responses to questions about the underlying rationale for initiating treatment were often vague, owners were very clear about one primary reason:

“*It was tough financially, very tough financially, and at that time I was advised to euthanise him. […] But it’s like...I think that when you choose to become a cat owner, or animal owner, you do it for life.*” (Julia)

“*But when it’s about it [medical treatment] being an inconvenience only for the owner that only causes like...Like, you have to engage daily with your animal. I think that you sign up for that when you take care of an animal.”* (Anna)

In these narratives, owners’ recognition of an enduring owner responsibility as a duty created when acquiring their cat was central. Assuming owner responsibilities as obligatory once the cat enters the human system of created dependency [26], individual cat-specific factors becomes less influential in decision-making, framing the choice of treatment as non-negotiable. Perceived obligations can serve as key motivators of owner care behaviours [27], which may be especially important when committing to the demands of DM treatment, at a stage when other motivators, such as enjoyment, are not yet evident. Many owners actively rejected opposing perspectives of responsible ownership that extends only until the cat’s health declines or the owner’s convenience dictates otherwise, demonstrating a notion of responsible ownership as incompatible with not doing all that could be done for the cat. This perspective challenges the societal norms that allow euthanasia to be based on owner convenience [28,29], emphasising a belief of ethical cat ownership entailing unwavering dedication, positioning responsibility as a moral imperative rather than a choice subject to external factors.

In the following, the three themes will be presented, starting with the reflections related to the very first information about their cat’s diabetic condition.

### Theme 1: In sickness and health

In the first theme generated, we focus on participants’ discussions of the motivation behind deciding upon medical treatment of their cat with DM, expanding on the perceived responsibilities of cat ownership. The certainty of selecting medical treatment was often likened to parenting, where the primary obligation was to ensure that the cat’s needs were met:

“*No, for me there was no doubt. She is my responsibility, like a child. If she had needed insulin every morning and evening, I would have solved it.*” (Hanna)

For Hanna, as for many participants, the cat was perceived as an integral family member, not merely in a linguistic sense. This seemed to intensify both the perceived bond and the moral commitment to provide DM care, with owners investing in treatment similar to how they would for immediate family. In previous research, categorisation of the cat as a child has been associated with a more caretaking and dependent relationship [1], and Anna’s account accentuates how the needs of her cat weigh heavier than any personal sacrifices. Applying an anthropomorphic view on the human-cat relationship is common [1,30,31], with multiple factors influencing owners’ conceptions of their animal [7,32–34]. By asserting their strong connection with their cat as a rational reason for pursuing DM treatment, owners framed medical care as an extension of the emotional bond. This was further presented in accounts where the type of relationship that owners seek shaped their perceived responsibility. Sofia reasoned “*some people might get a cat because they live on a farm and need someone to chase the mice away. They might not acquire a family member.”* It has been suggested that when a cat is seen as part of the household, it is considered an equal companion [1], and seeing the cat as a family member may intensify the moral commitment to choose treatment. Other participants framed their decision to treat DM by pointing out an irreplaceable inter-species bond:

“*I know that there are others who believe that when veterinary costs become too high, it’s time to say goodbye. […] I think they may be missing something—there’s a unique quality in the contact with an animal that they overlook. It’s somewhat special that you can connect with someone who looks so different from yourself, and cannot talk.*” (Anna)

Anna’s point of departure was that the owner–cat relationship offers unique emotional and psychological benefits, and she emphasized that this connectedness is created through interaction rather than simply given. Valuing the cat both for its intrinsic being and for the shared experiences and mutual responsiveness that develop over the course of ownership illustrates how humans construct meaning around their relationships with animals [35]. When cats are appreciated for providing support and company [36], and attributes such as “love” or “personality” [37], the strength of this bond can become central in owner reasoning about treatment [38]. In this light, owners with a long and positive relationship may feel a strong moral obligation to provide care after a DM diagnosis, whereas a weaker relationship may make the demands of treatment appear disproportionate to the perceived commitment:

*“[…] If I’d had her since she was a kitten, I would have felt differently. But when you have an animal that you’ve only had for a couple of months, and she’s been running and hiding all the time... If I’d been allowed to decide for myself, I’d probably have put her down there and then.”* (Ingrid)

By contrasting two situations, Ingrid illustrates how emotional bonds develop over time, and how a newly acquired animal lacks the shared history that may constitute the foundation of the owner-cat relationship. Here, the cat may be seen as a temporary responsibility rather than a long-term companion, making euthanasia a more immediately acceptable option. How the bond with the cat is experienced is shaped by the individual and their needs within the relationship [39]. Differing needs and levels of attachment among family members and bonds lacking reciprocity may therefore influence interpretations of responsibility [40]. This owner further explained that what was central in the decision to not euthanise the cat was the pronounced value the cat had held for her former partner. In this context, the cat’s perceived value became intertwined with her partner’s emotional needs and his sense of responsible ownership, indicating that the social and emotional context of decision-making may supersede individual preferences.

Other accounts of how care was negotiated within household dynamics was when caregivers had different thresholds for what they believed was in the cat’s best interest. Here, Marie shares how strong but differing emotional commitments within a social unit led to challenges in the decision-making in the early phases of treatment following her cat’s DM diagnosis:

“There were moments when I felt, ‘Damn, this isn’t working. This will never work.’ But he [the cat] is his dad’s cat—they are this *gesturing with pinched fingers, emphasising closeness*—and that is why he is still alive.” (Marie)

The owner’s admission of doubt of the benefits of medical care for the cat suggests that, independently, her threshold for discontinuing treatment may have been lower. The fear that extending treatment may come at the expense of the cat’s quality of life aligns with research showing that quality of life considerations serve as a key predictor of the decision to consider euthanasia [41]. While temporary discomfort may be justifiable if recovery is likely, the point at which medical intervention ceases to serve the animal and instead fulfils the caregiver’s needs has been recognised as an animal welfare concern [42].

These accounts demonstrate that decisions on treatment are negotiated within a social and emotional context of varying perceptions of owner responsibilities and acceptance of DM management. The owners’ discussions about their motivations for pursuing medical treatment encompassed the strong emotional and ethical considerations involved in caring for their cats with DM. However, as the narrative shifted to the cat’s experience as an individual living with chronic illness and undergoing treatment, other perspectives were constructed, reflecting the cat’s dual identity as both a beloved companion and an animal with specific medical needs.

### Theme 2: The cat: Opportunities and responsibilities

The second theme focused on the narrative of the cat as the subject of chronic disease and ongoing medical treatment. While care for the cat was still shaped by its status as a family member, a clearer distinction was made regarding the cat’s nature. This distinction provided an opportunity for a more flexible approach to medical care, with owners framing deviations from the desired medical regimen as a pragmatic alternative to euthanasia: *“I do my best, and that’s the way it is. […] It’s a bit like…You get two extra years. Because otherwise, she would have been euthanised.”* (Karin). The ability to adapt treatment routines to fit the owner’s lifestyle, the acceptance of some level of imperfection, and the focus on manageable care reflects a practical approach to DM management.

However, it was clearly emphasised that there was more to it than just making the cat survive, and weighing the quality of life of the cat against the burdens of disease and treatment was important. Across accounts, cat wellbeing and quality of life functioned as a guiding principle in decisions: “*My first question for the vet was about her quality of life; whether it was right to keep her, what was the right thing to do. It was important for me not to keep her alive just for my sake.”* (Amelia). It has been argued how owners, because of their physical and psychological proximity to their animal, may be or consider themselves better prepared to assess welfare than their veterinarian [43,44]. By actively seeking guidance and inviting the veterinarian into the decision-making process, Amelia positions herself somewhere between delegating the decision and making an autonomous decision, while emphasizing quality of life as a prioritized topic in owner–veterinarian communication [43,45]. In their concept of quality of life, owners listed appetite, the cat’s ability to live its desired lifestyle, and preserved interactions with humans, in accordance with previous reports [10,46,47]. Across accounts, owners were highly aware of their active role in management of DM and ensuring quality of life of their cat. Some owners were able to integrate the desired DM management plan into their lifestyle with ease, which was also described as a factor favouring the decision to initiate treatment. Fredrik described: “It’s just our routine at five o’clock when I get up—well, then there’s the injection and blood sampling and food. And then when I get home, there’s the injection…*laughs*”. Others found themselves having to make changes with treatment to be able to proceed, for reasons both related to the cat and to themselves and their day-to-day life:

*”If it had just been one long ordeal for her, if she had been stressed every day when I had to give her insulin, or if I had had to measure her blood sugar twice a day, it would not have worked. Not for her, or for me.”* (Mia)

*“If it had to be exactly 6 a.m. and exactly 6 p.m.. Then it wouldn’t have worked. So, you have to be able to slack a bit, like an hour here or there. You can’t be too hard on yourself, especially when you work and have small children.”* (Eva)

Supporting the rationale for these adaptations was the notion that not treating would lead to euthanasia, a decision that, proposedly, conflicts with the view owners could have on themselves as responsible cat owners. Reframing the desired management protocols as negotiable served to reduce owner and cat stress and sustain treatment adherence [46], suggesting a morally justifiable middle path. Owners did not directly describe their own quality of life as reduced by their cat’s DM treatment. Instead, they tended to report experiencing changes and restrictions in lifestyle and daily routines. However, the constant awareness of the cat’s dependence also carried an emotional weight: “*Caring for someone and being responsible for their health, especially when they can’t communicate, is obviously very stressful*” (Marie). This was particularly intensified for owners living alone or serving as the primary caregiver in their household:

*“Syringes and blood sugar monitoring are fine. […] However, I find it difficult that everything depends on it. If I choose to do something else one evening, like stay longer at a friend’s house for dinner, I immediately feel guilty for not giving him his insulin.”* (Julia)

Julia speaks not only the conflict between caregiver responsibilities and the desire to maintain social life or spontaneity, but her account also demonstrates feelings of social isolation, which may further intensify the burden of care. How owners perceive and cope with the constant awareness that the cat’s wellbeing depends on their actions can be an important source of distress, particularly in the absence of support systems [48]. Caregiver burden among owners of chronically ill companion animals include the negative impact of care on owner stress levels, social life, and feelings of insufficiency related to animal care [49], which seem to align closely with owners’ experiences of caring for a cat with DM. Some owners adapted treatment regimens to remain one step ahead of potential medical emergencies that could occur in their absence, including withholding scheduled insulin injections or reducing the insulin dose to prevent hypoglycaemic crises:

*“I’d rather have her blood sugar a little too high than too low, because when I’m not home, I can’t do anything about it. Sure, there are people at home, but they’re not really familiar with how to manage it.”* (Charlotta)

Central to pragmatic decisions like this is the recognition of the cat as a non-human animal, which allows for deviations from treatment protocols. As Charlotta further explained: “*It’s a cat. It’s not like a baby that will live for a hundred years.*” This attitude can partly be explained by the difference between caring for children and cats with DM, where cats are typically middle-aged or older at the time of diagnosis [50], leading to a different perspective on their remaining lifespan and the goals of treatment. Deviations from strict treatment were often justified by the perception that the cat’s quality of life remained acceptable. Management thus became a balance between medical control and practical caregiving, with owners often prioritizing quality of life over strict regulation, particularly when the cat was perceived as stable [46,51].

Owners also described positive effects in their lives following their cat’s DM diagnosis. Most accounts emphasised strengthening the owner–cat relationship and building trust, confirming results from previous studies on owners of cats with DM [10,52], but fulfilment was also found in the act of caregiving: “*It’s a big achievement to turn things around, and I’m very proud of it…knowing you’ve helped someone feel better again*” (Anna). This aligns with research indicating that providing care to an animal in need is associated with positive psychological outcomes, such as increased feelings of being needed and valued [53]. Yet alongside this sense of fulfilment, reflections frequently turned to the difficult questions of how long treatment should continue and when euthanasia might represent the best decision.

### Theme 3: Who decides about life?

The third theme captured owners’ accounts as they reflected upon euthanasia decisions, where quality of life of their cat once again served as a central factor. This brought out deeper philosophical and moral questions: who has the right to end a life, and under what conditions should it be considered? Or as one owner explained: “*I don’t kill a spider inside; I carry it outside. Who am I to end a life?*“ (Karin). Euthanasia was framed as permissible only once a threshold had been crossed, loosely defined in terms of the cat’s welfare and quality of life:

*“I have promised him [the cat] that I will never euthanise because of my finances or my energy. […] I believe it should be about the cat’s well-being. When the cat is in pain or feels so unwell that it becomes a relief for the cat...Then I think it’s time. It’s easy to say that, but you don’t really know. And that’s where I am now. I don’t know if it would be a relief for him or not.”* (Julia)

As with the decision to initiate DM treatment, owners explicitly rejected convenience or financial motives as legitimate reasons. Julia’s account suggests that choosing euthanasia for any reason other than an unavoidable decline in quality of life would compromise her responsibility towards the cat. This perspective adds to previously recognized factors influencing euthanasia decisions, including quality of life [54,55] and the burdens of caregiving [41]. Determining what is in the cat’s best interest and weighing its suffering against its presumed will to live are fundamental moral questions in veterinary medicine [56,57]. What initially appears to be a rational assessment quickly becomes difficult as the realities of assessing perceived suffering and quality of life in the context of chronic disease emerge, and Julia finds herself without a clear answer. The challenge that a slow decline may bring, with a lack of a clear-cut endpoint, made many participants speak of the evaluation as emotionally taxing and constantly present. Even when deterioration in DM was evident, some owners experienced the decision as a moral breach:

*“It felt like things just kept getting worse and worse. So, we did it—we let him go. And of course, it was incredibly difficult. It feels a bit like a betrayal that I didn’t keep fighting, but at the same time...It felt like we had been trying for so long, and he wasn’t getting any better. […] We had reached the end of the road.”* (Hanna)

In this account, having made past commitments to sustain the cat´s life through the course of DM made the euthanasia decision increasingly burdened with guilt. This suggests that responsibility may be constructed as perseverance, and the tension lies between minimizing suffering (the cat’s welfare) and honoring a sustained duty of care (the owner’s commitment). Owners’ accounts of guilt and self-blame related to euthanasia decisions mirrors previous reports [41,58]. Such guilt may stem from the conflict of acting against one’s self-concept of responsible ownership [59], but it may also reflect the depth of the emotional bond between owner and cat. Some owners described a conflict between the lifestyle burden of treatment and euthanizing a cat that was otherwise doing well. The ethical and emotional weight of euthanasia led them to hope for a natural, unassisted death:

*“It would just be better if we came home one day, and she wasn’t alive. Not that she had fallen and hit her head, but that she just went to bed and didn’t wake up again. It’s a pretty horrible thought, really, but I feel it would be so much nicer not to have to make that decision. Because I’ve already made the decision to keep her, and I feel that I don’t want to decide on that again.”* (Ingrid)

The wish for a “natural” death may reflect an attempt to shift the moral burden of decision-making and the responsibility for the cat’s death away from the owner [60]. Perseverance again appears central, but in this context it raises questions about whether treatment must be continued indefinitely, particularly when it conflicts with maintaining a social life, starting a family, or simply being free from the constant concerns of care, as exemplified by some owners. Proposing cessation of treatment and euthanasia for a clinically stable cat is ethically fraught and can be hard to raise with veterinarians [43], particularly where owners and clinicians may disagree about the animal’s moral worth and the limits of caregiving. The preferred role of the veterinarian was clear to some owners, but preferences differed: either a shared decision-making process that respects client autonomy [61], or a more directive role in which the veterinarian influences and leads the decision-making [62], thereby simplifying choices. For example, one owner explained: *“I hope that when she gets pancreatitis at 18 years old, the veterinarian will say, ‘This is no longer feasible.’ Somehow. Because then I haven’t made that decision.”* (Eva).

## Summary and clinical importance

This study contributes to in-depth findings that expand the understanding of perceived responsibilities and emotional connections in owner decisions regarding medical treatment, quality of life, and end-of-life choices in cats with DM. We identified that owners’ sense of life-long obligation and the emotional bond with the cat influenced treatment decisions, making them prioritize treatment and care despite personal sacrifices. We also provide new insights into how DM management is actively negotiated within a social and emotional context, where varying acceptance of DM management and perceptions of owner responsibilities co-exist. The owners in this study situated themselves as invested in DM care, yet burdens of disease management and experience of social isolation were present. For the veterinary team, it is crucial to acknowledge the emotional toll of caring for a chronically ill cat and attention to owners’ experiences and perspectives is needed to improve communication. Recognising this, and the fact that individual owners may want varying degrees of veterinary involvement in decision-making and require different treatment approaches, could enhance compliance and overall owner well-being. We argue that the veterinary team would benefit from recognising the close bonds between owners and their cats and providing individualised care plans to improve veterinary support and cat welfare. Addressing both the medical and emotional aspects of caregiving is crucial for fostering a sustainable caregiving experience.

## Limitations and suggestions for future research

As cultural, gender-related, demographic, and other contextual factors influence reasoning [63], the composition of the group of owners naturally shaped the analysis and results. The contextual settings of the research must be considered when locating the findings in relation to other owner groups, species or circumstances. Care was taken to ensure that different aspects of participants, such as their gender, age, geographic location and the outcome for their cat (under insulin treatment, achieving remission, deceased), were considered. However, despite efforts, it proved difficult to recruit a more widely diverse range of participants; for example, 11 of 12 owners were female. This may reflect differences in decision-making between male and female owners in managing DM, where factors influencing these decisions could include a greater emotional investment in the cat observed among female owners [6,38], or a heightened sense of caregiving behaviour in women, as has been suggested [1]. Most participants were recruited via a Facebook group on cat DM, which may over represent highly motivated owners and limit transferability of the findings to the broader population of cat owners facing a DM diagnosis. Owners found themselves in different phases of DM management, and not all cats were alive at the time of the interviews. Although this contributed to a diversity of experiences and accounts, it is important to acknowledge that perceptions and sense-making of such experiences may change over time [55,64].

Focusing solely on the experiences of owners who chose to medically treat their cat diagnosed with DM might result in a group of caregivers more likely to report a positive caregiving experience compared to those who opted for euthanasia. On the contrary, the richness and complexity of the generated data reflect a depth and diversity within this group, highlighting that it is not homogenous but encompasses a range of experiences and viewpoints. Further, this selection of informants enables us to address a specific research question never investigated before. Previously found reasons for euthanasia upon DM diagnosis have reflected a poor prognosis, like concurrent diseases and the desire to spare the cat from suffering [15,17]. Other reasons include a too-great impact on the owner’s lifestyle and finances [15]. Our study shows that there is a willingness to take on this ‘duty of care’, while in-depth information on owners’ reasoning behind the decision to euthanise is lacking. Therefore, future research should qualitatively explore the perspectives of owners who chose not to treat their cats with DM.

## Supporting information

S1 Appendix Additional information on methodological and analytical considerations. (DOCX)

S2 Appendix Interview guide. (DOCX)
