# Supplementary material for: In sickness and health: A reflexive thematic analysis of ethical considerations and experiences of owners of cats treated for diabetes mellitus
Source: PLoS One. 2026 Feb 2;21(2):e0341759. doi: 10.1371/journal.pone.0341759 (PMC12863498; doi:10.1371/journal.pone.0341759)
Supplement: S1 Appendix — (DOCX) [file pone.0341759.s001.docx]

**S1 Appendix. Additional information on methodological and analytical considerations.**

Here, additional information on methodological considerations and procedures is provided.

Veterinary practice is inherently complex; not only in terms of medical care and clinical decision-making, but also due to the human–animal relationship, and the interactions between veterinarians and owners. To gain a deeper understanding of complex issues, including what drives human behaviour and how individuals interpret clinical situations, it is essential to consider their perspectives. Qualitative research is well suited both for exploring complex phenomena and for deepening the understanding on a given subject, including the examination of experiences, perceptions, and the nuanced dimensions of human behaviour (1).

**Methodological framework**

Decision-making is influenced not only by the perception of a specific situation (e.g., a veterinary visit), but also by prior experiences, upbringing, and the socialisation surrounding a phenomenon (e.g., views on animals and on life). Subjective and social experiences shape an individual’s reality, and there is no single version of how to experience a situation. This subjective world must be interpreted to gain a deeper understanding and exploration of a specific phenomenon (2). Based on this reasoning, the research questions were generated within a constructivist framework, aiming at faithfully presenting the lived experiences and rich meanings of the participants. Applying an experiential orientation towards the data allowed for the highlighting of individual and diverse experiences, granting participants a central role in shaping the narrative. For the researcher, this implies the need to delve deeply into the specific context, striving to provide nuanced and situated understandings of the subject. Given the research questions and the underlying paradigmatic assumptions, as well as the presumed complexities associated with the subject, the research team reasoned that the study would benefit from a reflexive approach (3, 4). In this way, the subjectivity of the authors' own experience and knowledge of the topic enriched the analytic process, and allowed data collection and analysis to ensure participant subjectivity in the matters discussed during the interviews (3).

**Interview guide and interviews**

To capture the depth and complexity of these lived experiences, and to enable the exploration of lines of reasoning, flexibility in questions and follow-up enquiries is necessary. This includes the ability to ask participants to elaborate on or provide examples of specific situations or thought processes (4, 5). Therefore, individual in-depth interviews were selected for data collection. The interview guide was piloted with two cat owners, both female, who had experience choosing to medically treat their cat following a diagnosis of diabetes mellitus (DM). At the time of the pilot interview, one owner’s cat was alive, whereas the other owner’s cat had been euthanised. The pilot interviews were video recorded and reviewed by the first author and a co-author to assess question clarity, comprehension, and relevance to both participants and the research aim. The review also considered whether the questions allowed participants to engage with the topic in a manner appropriate for the methodological approach. Based on this assessment, adjustments were made to the interview guide. For example, the original question, “Could you describe your cat’s treatment regimen?” was revised to “What was important to you when making decisions about treatment?” to better capture the underlying rationale and to allow participants to emphasise their experiences and factors that influenced their treatment decisions. While all questions in the guide were addressed during interviews, the order of questioning varied among interviews, following the flow of topics and argumentation lines. Probing follow-up questions were used as needed.

The interviews were conducted in Swedish, the majority through a digital video conference program (Zoom). Verbal consent from participants to allow the recording of the interviews was documented in writing by the interviewer (the first author). Since video recording began only after consent was obtained, the consent itself was not recorded. However, participants were notified via Zoom that a recording had started, were informed about the recording icon and its meaning, and this was **documented on video.** Participants had also received prior information about the recording of the interviews, giving them time to consider participation. One participant did not wish to be video recorded; therefore, only audio was recorded. To minimise the risk of power asymmetry during the interviews, significant attention was given to establishing good rapport (6). By ensuring a safe and supportive interview environment for the owner and by providing clear information about the first author, the study and its purpose, the likelihood that the interviewees would share their true perspectives increased. This reduces the risk of opt-outs and of variability in responses and ascribed meanings (5).

To determine and evaluate the number of interviews, data collection and the information obtained were assessed stepwise and continuously. This process was guided by the concept of information power (7), where the level of richness and the relevance of information in the dataset informs the number of participants, and Braun and Clarke’s reflections on data collection within RTA (8). The exploratory approach and the broader aim of the study, the few inclusion criteria, and the analytic strategy (RTA) indicated the need to include a larger number of participants. In contrast, the richer data items (in-depth interviews) and the study being theoretically informed (e.g., previous studies on human-animal relations and the research group´s experience in the field), generally entail fewer participants. An initial set of five interviews was conducted consecutively. During these, field notes were taken to review the richness and quality of the data and the variety of participants’ experiences. Interviews continued, and the new data were assessed in relation to the initial five and the following interviews, with a focus on data quality and their relevance to the research questions. After ten interviews, the dataset was judged to be sufficiently rich. However, given the explorative nature of the study, two additional interviews were conducted to avoid prematurely closing data collection and reduce the risk of limiting sample specificity regarding participants’ experiences. The recorded video interviews were transcribed manually and verbatim by the first author, applying consistent syntax for non-verbal occurrences (e.g. laugh, nod) to enhance transcription quality (9). The software oTranscribe was used. To prevent undue influence from already collected data and to maintain focus during subsequent interviews, interview transcription was not finalised until all interviews had been completed.

**Analytical process**

A six-phase, inductive reflexive thematic analysis (RTA) was undertaken to systematically interpret meanings and explore patterns across the dataset (3, 10), see Figure 1. Different phases of the analysis required different approaches to the data. Accordingly, the qualitative analysis software Delve was used primarily during the initial coding, refining of codes, and structuring of the data, while printed copies of the transcripts and codes were used in later phases when another visualization was needed for theme development. Each step of the analysis was documented. First, NRZ (the first author) read the transcripts to become familiar with the participants’ individual experiences, annotating preliminary observations and potential points of analytical interest. The transcripts were then systematically and iteratively examined, and data excerpts of interest and relevant to the research questions were coded in an inclusive manner. Surface level coding was applied to clarify what participants had expressed and an interpretive lens was used where deeper understanding was warranted, especially in discussion points where participants might have had difficulty expressing themselves. As the analysis progressed, coding shifted from a predominantly semantic focus toward a greater emphasis on latent content. To promote reflexivity and encourage critical engagement, two transcripts were independently coded by both the first author and a co-author, serving as a basis for subsequent discussions and reflections on the data.


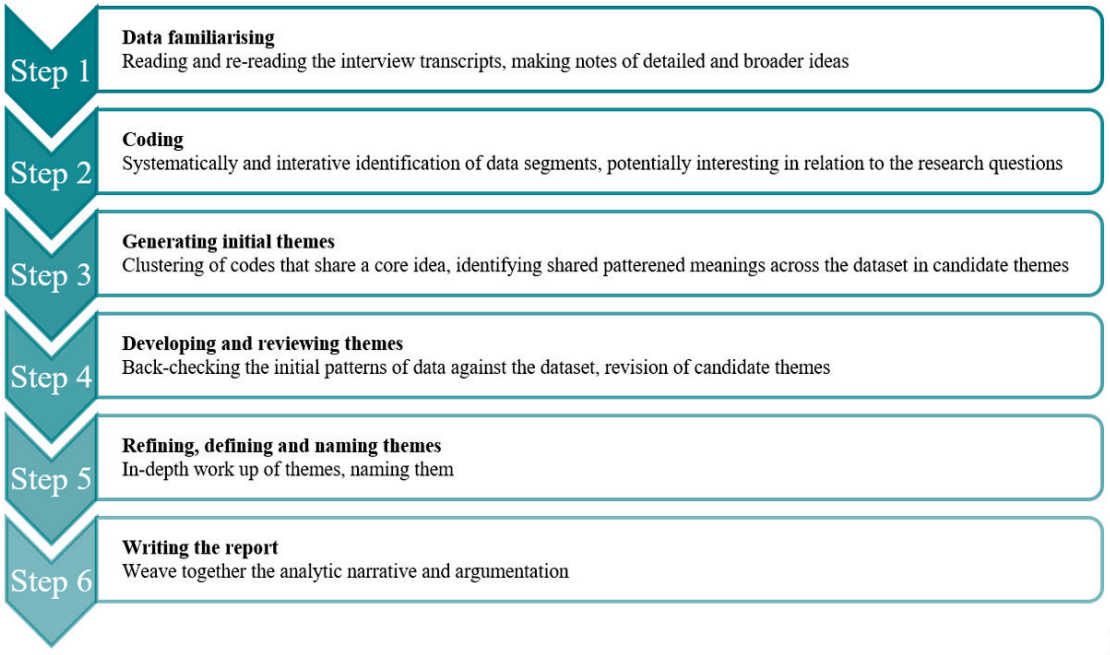


**Figure 1.** Overview of the analytical process in RTA. Note that the process is not linear, and revisiting earlier phases are important steps in analysis and for fostering researcher reflexivity.

Codes were iteratively refined and elaborated upon through ongoing analysis. Related codes were merged and new codes were developed and added where relevant. In the next phase, codes were clustered into broader patterns of shared concepts and meaning (candidate themes): owners’ motivations to treat, how owners related to ownership and to the cat as a receiver of care, and end-of-life decision-making. The candidate themes were reviewed against their underlying codes and supporting data extracts to ensure accurate representation, with openness to revision where needed. Discussions among the authors guided the final development of themes, and, as it became apparent that ownership itself served as a broader conceptual framework encompassing reasoning across the dataset, one over-arching theme. Summarising the definitions and narrative of each theme served to both check and clarify the shared concept of included data and to decide on the structure and order of theme reporting. At this stage, engagement with existing research situated the data and deepened the analysis. Through the analytical course, regular discussions within the research team of meanings, codes, themes and patterns in the data served to draw upon the diverse perspectives and experiences, in order to enhance and broaden our analytical insights.

As an example of the analytical process, the data extract “*To euthanize an animal that isn't sick enough that you have to do it, that just feels wrong to me.*” (Mia), referring to a euthanasia decision not directly prompted by deterioration due to DM, was initially coded as “Euthanasia only when necessary.” Realizing this code was too vague and did not adequately reflect the owner’s emotional struggle, it was later revised to “Justification of euthanasia upon disease”, interpreted as a value-laden statement expressing discomfort with making an end-of-life decision when the cat is not critically ill. Upon revisiting the dataset, the code was further refined to “Values guiding euthanasia decisions”, broadening its scope to include related extracts articulating ethical and emotional frameworks surrounding euthanasia decisions. This code was initially grouped, alongside codes of related and shared meaning, under the candidate theme “A principled choice” to reflect the owner's active role in decision-making, shaped by personal ethics. The theme was finalized as “Who decides about life”, capturing the broader idea of euthanasia as emotionally and ethically challenging.

| Statement | Iterative coding process | | | Candidate theme | Theme |
| --- | --- | --- | --- | --- | --- |
| “*To euthanize an animal that isn't sick enough that you have to do it, that just feels wrong to me.*” | Euthanasia only when necessary | Justification of euthanasia upon disease | Values guiding euthanasia decisions | A principled choice | Who decides about life |

**Table 1**. Example of the inductive analytical process.

**Reflexivity**

In RTA, the researcher is understood as inseparable from the research process, with their interpretations, choices and positioning shaping research practices, making it inherently subjective. Discussions in the research team focused on deepening interpretations rather than achieving coding agreement, aiming to capture a broader range of perspectives rather than obtaining a more “accurate” representation of the phenomenon. The reflexive process, which includes reflections on researcher positionality and how it may influence data engagement (3), was primarily informed by NRZ, a female PhD student with training in conducting qualitative research. NRZ’s clinical experience as a veterinarian with experience in treating DM informed the interpretive process, while ongoing discussions with co-authors supported reflexivity and contextual awareness. Familiarity with the clinical and relational complexities of DM in cats served as a valuable resource (11), helping to resonate with participants’ accounts. To foster reflexivity and acknowledge how NRZ’s active role and prior experiences shaped the generation of knowledge, ongoing reflection was undertaken throughout the analytical process. This included considering what aspects of the data elicited particular interest, and how personal context and professional background influenced that response, with deliberate effort to remain open to owners’ experiences and to explore their reasoning without preconceived assumptions. After initial coding, sparsely coded sections were revisited to ensure inclusivity and to avoid overemphasizing areas of personal relevance. Discussions with co-authors supported this reflexive approach by encouraging openness to alternative interpretations and a willingness to let go of narrow or preconceived analytical frames.

**References**

1. Pope C, Mays N. Qualitative Research: Reaching the parts other methods cannot reach: an introduction to qualitative methods in health and health services research. BMJ. 1995;311(6996):42-5.

2. Höglund Nielsen B, Granskär M. Tillämpad kvalitativ forskning inom hälso- och sjukvård: Studentlitteratur; 2017.

3. Braun V, Clarke V. Reflecting on reflexive thematic analysis. Qualitative Research in Sport, Exercise and Health. 2019;11(4):589-97.

4. Braun V, Clarke V. Conceptual and design thinking for thematic analysis. Qualitative psychology. 2022;9(1):3.

5. Kvale S, Brinkmann, S. Interviews: Learning the craft of qualitative research interviewing: SAGE Publications Inc.; 2009.

6. Dicicco-Bloom B, Crabtree BF. The qualitative research interview. Medical Education. 2006;40(4):314-21.

7. Malterud K SV, Guassora AD. Sample Size in Qualitative Interview Studies: Guided by Information Power. Qualitative Health Research. 2016;26(13):1753-60.

8. Braun V, Clarke V. To saturate or not to saturate? Questioning data saturation as a useful concept for thematic analysis and sample-size rationales. Qualitative Research in Sport, Exercise and Health. 2021;13(2):201-16.

9. Poland BD. Transcription quality as an aspect of rigor in qualitative research. Qualitative inquiry. 1995;1(3):290-310.

10. Braun V, Clarke V. Using thematic analysis in psychology. Qualitative Research in Psychology. 2006;3(2):77-101.

11. Gough B, Madill A. Subjectivity in psychological science: from problem to prospect. Psychological methods. 2012;17(3):374.
