# Supplementary material for: In sickness and health: A reflexive thematic analysis of ethical considerations and experiences of owners of cats treated for diabetes mellitus
Source: PLoS One. 2026 Feb 2;21(2):e0341759. doi: 10.1371/journal.pone.0341759 (PMC12863498; doi:10.1371/journal.pone.0341759)
Supplement: S2 Appendix — (DOCX) [file pone.0341759.s002.docx]

**S2 Appendix. Semi-structured interview guide.**

You have or have had a cat with diabetes. Would you like to tell me about your cat?

How would you describe your relationship with your cat?

- How much time do you spend together/what do you do when spending time together/how much does your cat mean to you?

Do you remember when your cat was diagnosed with diabetes mellitus?

- How did you feel at that time?

What were your thoughts when deciding upon your cat’s treatment?

What was important to you when making decisions about treatment?

How did the collaboration with the veterinarian work? Did you agree on the treatment plan?

What happened next (after the diagnosis)?

How do/did you feel about the treatment?

How have you been affected by your cat’s diabetes?

- Can you think of anything about the diabetes mellitus that has affected you and your cat positively?
- And negatively?
- How would you say that your relationship with your cat has been affected by the diabetes mellitus?

What would you say constitutes a good quality of life for a cat?

Can you recall a time when you were concerned about your cat’s quality of life?

When do you think euthanasia is an acceptable decision for a cat with diabetes?

- When is it not?
- What determines when the time is right?

(If applicable) Euthanasia after a period of treatment:

- Do you remember what happened?
- Do you remember what led you to the decision to euthanise?
- Was it something you/your family had been thinking about, or was it a sudden decision?
- How did you know it was time?
- Were you and the veterinarian in agreement?
- Have you experienced the euthanasia of a cat before?
- How do you feel about it today?

What do you think the veterinarian’s role should be?

- How was your veterinarian?

*Please note that, due to the semi-structured nature of the interviews, additional questions, probing, and follow-ups were incorporated as needed. Furthermore, the order of the questions in the guide is intended to be only a guideline and was adjusted to follow the natural flow of each interview.*
